# Exosomal miR-132-3p from mesenchymal stromal cells improves synaptic dysfunction and cognitive decline in vascular dementia

**DOI:** 10.1186/s13287-022-02995-w

**Published:** 2022-07-15

**Authors:** Xiaotang Ma, Yan Wang, Yumeng Shi, Suqing Li, Jinhua Liu, Xiangyong Li, Wangtao Zhong, Qunwen Pan

**Affiliations:** 1 Department of Neurology, Guangdong Key Laboratory of Age-Related Cardiac and Cerebral Diseases, Affiliated Hospital of Guangdong Medical University, Zhanjiang, 524001 China; 2 Institute of Biochemistry and Molecular Biology, Guangdong Medical University, Zhanjiang, 524001 China

**Keywords:** Vascular dementia, Mesenchymal stromal cells, Exosomes, miR-132-3p, Synaptic plasticity

## Abstract

**Background/aims:**

Vascular dementia (VD) results in cognition and memory deficit. Exosomes and their carried microRNAs (miRs) contribute to the neuroprotective effects of mesenchymal stromal cells, and miR-132-3p plays a key role in neuron plasticity. Here, we investigated the role and underlying mechanism of MSC EX and their miR-132-3p cargo in rescuing cognition and memory deficit in VD mice.

**Methods:**

Bilateral carotid artery occlusion was used to generate a VD mouse model. MiR-132-3p and MSC EX levels in the hippocampus and cortex were measured. At 24-h post-VD induction, mice were administered with MSC EX infected with control lentivirus (EX^Con^), pre-miR-132-3p-expressing lentivirus (EX^miR-132-3p^), or miR-132-3p antago lentivirus (EX^antagomiR-132-3p^) intravenously. Behavioral and cognitive tests were performed, and the mice were killed in 21 days after VD. The effects of MSC EX on neuron number, synaptic plasticity, dendritic spine density, and Aβ and p-Tau levels in the hippocampus and cortex were determined. The effects of MSC EX on oxygen–glucose deprivation (OGD)-injured neurons with respect to apoptosis, and neurite elongation and branching were determined. Finally, the expression levels of Ras, phosphorylation of Akt, GSK-3β, and Tau were also measured.

**Results:**

Compared with normal mice, VD mice exhibited significantly decreased miR-132-3p and MSC EX levels in the cortex and hippocampus. Compared with EX^Con^ treatment, the infusion of EX^miR-132-3p^ was more effective at improving cognitive function and increasing miR-132-3p level, neuron number, synaptic plasticity, and dendritic spine density, while decreasing Aβ and p-Tau levels in the cortex and hippocampus of VD mice. Conversely, EX^antagomiR-132-3p^ treatment significantly decreased miR-132-3p expression in cortex and hippocampus, as well as attenuated EX^miR-132-3p^ treatment-induced functional improvement. In vitro, EX^miR-132-3p^ treatment inhibited RASA1 protein expression, but increased Ras and the phosphorylation of Akt and GSK-3β, and decreased p-Tau levels in primary neurons by delivering miR-132-3p, which resulted in reduced apoptosis, and increased neurite elongation and branching in OGD-injured neurons.

**Conclusions:**

Our studies suggest that miR-132-3p cluster-enriched MSC EX promotes the recovery of cognitive function by improving neuronal and synaptic dysfunction through activation of the Ras/Akt/GSK-3β pathway induced by downregulation of RASA1.

**Supplementary Information:**

The online version contains supplementary material available at 10.1186/s13287-022-02995-w.

## Introduction

Vascular dementia (VD), a degenerative cerebrovascular disorder resulting in cognitive impairment and memory decline, is currently the second leading form of dementia (at least 20%) following Alzheimer’s disease (AD)。However, there are still no available treatments to slow or reverse the damage caused by VD [1]. Similar to AD and other cognitive disorders, neuronal damage and synaptic dysfunction in the hippocampus and the cortex are the main causes of VD [1]. Therefore, protection of neuronal density, synapse formation, and synaptic plasticity are important for ameliorating cognitive impairment after VD.

Mesenchymal stromal cells (MSCs), which can be conveniently isolated from various tissues, have shown potential therapeutic effects on neurological diseases and injury through neuronal differentiation and paracrine action [2, 3]. Recent studies suggested that the paracrine activity, rather than differentiation, played a vital role in the protective effects of MSCs [4, 5]. Exosomes (EX), a nanosized membrane vesicles secreted by most cells, may affect recipient cell function by transferring their cargos, including protein and microRNAs (miRs), which are considered a novel tool for stem cell paracrine action [6]. In the present study, EX was isolated from bone marrow MSCs, which with the abilities to differentiate into adipocytes, chondrocytes, osteocytes, and can also promote tissue homeostasis following injury [7]. Moreover, bone marrow MSCs are currently used for therapeutic purposes and are under scrutiny in numerous clinical trials for the treatment of several human diseases [8]. However, the isolation of MSCs according to International Society for Cellular Therapy (ISCT) produces heterogeneous, non-clonal cultures of stromal cells containing with different multipotential properties, committed progenitors, and differentiated cells [7, 8]. Although the nature and functions of MSCs remain unclear, non-clonal stromal cultures obtained from bone marrow that contain a subpopulation of stem cells are currently serving as sources of putative MSCs for therapeutic purposes [9]. Several studies had demonstrated that EXs derived from MSC (MSC EX) exerted therapeutic effects on neural impairment in various neurological diseases, such as ischemic stroke and AD [6, 10]. In an AD mouse model, systemic administration of MSC EX ameliorated cognitive decline by decreasing Aβ accumulation and synaptic dysfunction [11]. However, there are no reports demonstrating whether MSC EX can protect neuronal damage and cognitive decline resulting from VD.

Studies have shown that the therapeutic effects of MSC EX are highly correlated with their miR cargos. Therefore, the use of EXs engineered to carry neuronal protective miRs for the treatment of neurological diseases represents a promising strategy. Chopp et al. reported that miR-17-enriched MSC EX improved neurological outcome by increasing neurogenesis and neurite outcome in a rat stroke model [12]. MiR-132-3p is one of the most abundant miRs in brain tissue and has been shown to exert a strong neuroprotective effect [13]. In the hippocampus, a reduction of miR-132-3p was associated with decreased spine density, reduced synapse formation, increased Aβ production, and Tau hyperphosphorylation [14, 15]. In primary cultured neurons, miR-132-3p inhibition induced cell apoptosis via the PTEN/Akt signaling pathway. Furthermore, studies had shown that miR-132 promoted axon outgrowth by repressing the targeted gene, Rasa1, and activating the Ras signaling pathway [16, 17]. The Ras/Akt/GSK-3β signaling pathway has been found to be implicated in the regulation of axonal outgrowth and neuronal function recovery [18]. Thus, we hypothesize that enrichment of miR-132-3p may increase the beneficial effects of MSC EX on VD-induced neuron damage and cognitive impairment.

In this study, we investigated the effects of miR-132-3p-enriched MSC EX on neuron damage, synaptic dysfunction, and cognitive impairment in a VD mouse model and the underlying mechanism of action by analyzing the Ras/Akt/GSK-3β signaling pathway.

## Materials and methods

### Generation of miR-132-3p enriched or knock down MSC exosomes

Mesenchymal stromal cells (MSC) were cultured from bone marrow of C57BL/6 mice as we previously described [19]. Control MSC exosomes (EX^Con^), miR-132-3p-enriched MSC exosomes (EX^miR-132-3p^), and miR-132-3p knockdown MSC exosomes (EX^antagomiR-132-3p^) were collected from MSC transfected with scrambled control (MSC^Con^), lentivirus carrying the murine miR-132-3p (MSC^miR-132-3p^), or lentivirus carrying the murine antago miR-132-3p (MSC^antagomiR-132-3p^), respectively.

## MSC proliferation assay and senescence assay

Proliferation capability of MSC was tested by Cell Counting Kit-8 (CCK-8) based on manufacturer’s instructions and a recent study described [20]. We seeded 4000 cells in 96 wells and CCK-8 were added. The viability of MSC^Con^, MSC^miR-132-3p^, or MSC^antagomiR-132-3p^ was detected by a microplate reader at 450 nm 24 h, 48 h, and 72 h after the incubation.

MSC senescence was determined with a Senescence β-Galactosidase Staining Kit (Beyotime, China) as we previously described with minor modifications [21]. In brief, after washing with PBS, MSC^Con^, MSC^miR-132-3p^, or MSC^antagomiR-132-3p^ was fixed for 6 min in 2% formaldehyde and 0.2% glutaraldehyde and then incubated for 12 h at 37 °C with fresh X-gal staining solution. The percentage of senescent cells was calculated by the number of blue-stained cells (β-Galactosidase-positive cells) out of total cells in different microscope fields.

### Animals

Adult (8–12 weeks old) male C57BL/6 mice were purchased from the Animal Experiment Center of Guangdong Province (Guangzhou, China) and raised in the animal care facility at the Guangdong Medical University. The mice were housed in a pathogen-free environment. Surgeries were performed under 2.5% isoflurane anesthesia. All experimental procedures were approved by the laboratory animal care and use committees at Guangdong Medical University.

### EX infusion of mouse VD model

The VD mouse model was generated as previously described [22]. Briefly, mice were anesthetized with isoflurane, fixed on a stereotaxic instrument, and body temperature was maintained at 37 °C with a feedback-regulated water heating system. A skin incision was made in the neck area, the bilateral carotid arteries were separated and locked by ligation for 10 min and then released for 10 min. This process was repeated three times. The sham-operated mice (Control) underwent the same procedure except the bilateral carotid arteries were locked. Twenty-four hours after VD, the mice were administered PBS (vehicle), EX^Con^, EX^miR-132-3p^, or EX^antagomiR-132-3p^ (1 × 10^10^ particles/100 μl) via the tail vein once every 7 days for 21 days. This dose was selected based on previous studies on MSC EX administration for treating CNS diseases [23]. Memory and cognitive performance were measured by the Morris water maze (MWM) test on the last 5 days of the experiment. Immediately after the MWM test, the mice were killed at day 21 following MSC EX infusion, and the cortex and hippocampus were collected and used for various measurements including NTA analysis, immunofluorescence, immunohistochemistry, and Golgi staining.

### Detection of MSC EX in brain tissue

MSC EX was isolated from the cortex and hippocampus of Sham (MSC EX^Sham^) or VD (MSC EX^VD^) mice as described previously with some modifications [24]. Briefly, the cortex and hippocampus were separated and finely minced with small, sharp scissors in 100 μl of papain solution (20 units/ml). Brains in solution were pipetted into a 15-ml conical tube containing 3.5 ml papain solution and incubated for 20 min at 37 °C to dissociate the tissue and free the extracellular space. Then, the total brain EXs were isolated using sequential centrifugation at 300×*g* for 10 min, 2000×*g* for 20 min, 20,000×*g* for 90 min, and 100,000×*g* for 2 h after the supernatant was passed through a 0.22-μm syringe filter (Millex-GP, Millipore). The pelleted EXs were incubated with 10 μl of biotin-conjugated anti-CD29 antibody (Miltenyi Biotec) in a 100-μl reaction volume for 2 h, followed by the addition of 10 μl of anti-biotin microbeads for 15 min. The microbeads labeled EX from the total brain EX suspension were separated with a DynaMag-2 magnet (Life technology). After an overnight magnetic separation, the microbead-bound EXs were collected and resuspended in 100 μl of particle-free PBS. The suspension was incubated with 10 μl multisort release reagent (Miltenyi Biotec) to cleave off the microbeads. The EX in the fluid was collected and considered MSC EX (CD29^+^ EXs). The pelleted MSC EX was resuspended in phosphate-buffered saline (PBS) and aliquoted for nanoparticle tract analysis (NTA), transmission electron microscopy, and EX-specific marker CD63 and MSC-specific marker CD29 measurement using western blot analysis.

### Detection of MSC EX interacting with neurons in the cortex and hippocampus

MSC EX was labeled with PKH26 (Sigma) and resuspended in PBS for infusion [11]. After 24 h of infusion, the brains were dissected from mice, frozen in liquid nitrogen, and cut into 20-μm-thick sections. The brain sections were incubated with rabbit monoclonal anti-NeuN antibody (1:300; Abcam) at 4 °C overnight, and the sections were then incubated with goat anti-rabbit IgG H&L (Alexa Fluor® 488) secondary antibody for 1 h. After rinsing with wash solution, the interaction of MSC EX with neurons in the cortex and hippocampus was detected by confocal microscopy (Olympus Corporation, Japan).

### Analysis of miR-132-3p level

The levels of miR-132-3p in MSC, MSC EX, neurons, cortices, and the hippocampus were measured by the stem-loop reverse transcription (RT)-based TaqMan assays. Total miRs from mice and cells were isolated using the miRNeasy Mini kit (QIAGEN) based on the manufacturer’s instructions. The miR-132-3p cDNA was synthesized using the Hairpin-it™ microRNA and U6 snRNA RT-PCR Quantitation kit (GenePharma, Shanghai, China) under 25 °C for 30 min, 42 °C for 30 min, and 85 °C for 5 min. The generated cDNA was used for real-time PCR analysis (Roche 480). Samples were analyzed in triplicate, and U6 was used as an internal control. The primer sequences used are listed below: 5-CCAGCATAACAGTCTACAGCCA-3 and 5-AACGCTTCACGAATTTGCGT-3 for miR-132-3p, and 5-CTCGCTTCGGCAGCACA-3 and 5-AACGCTTCACGAATTTGCGT-3 for U6. A total reaction mixture of 10 μl was amplified in a 96-well PCR plate (BIOplastics, Netherlands) using the following cycling conditions: 95 °C for 3 min followed by 40 cycles of 95 °C for 12 s and 60 °C for 40 s. The relative quantification of the gene expression was determined using the comparative CT method (2^−△△Ct^).

### Morris water maze test

The labyrinth was composed of a barrel water tank (120 cm in diameter with a platform filled with tap water at a temperature of 22 ± 2 °C). An escape platform with a diameter of 10 cm was placed 1–2 cm below the water surface; mice were placed into the maze at one of four points (N, S, E, W) facing the wall of the tank. Over a period of five days, the mice were allowed to find the platform for 90 s. If the mice failed to find the platform, it was guided to the platform and allowed to rest for 15 s. On day five, the platform was removed and a probe test was conducted. The percentage time spent in each of the four quadrants and the number of target area crossings, total distance, and mean speed were recorded.

### Immunohistochemistry

The number and arrangement of neurons in hippocampal CA1 region were detected by immunohistochemical staining. Each group of brain tissue was fixed in 4% formalin for 24 h, and then, the samples were dehydrated in serially graded ethanol solutions, defatted in methanol and embedded in paraffin. The brain tissues were coronally sectioned at a thickness of 6 μm, deparaffinized in xylene, and rehydrated in descending concentrations of alcohol. Endogenous peroxidase activity was blocked with 3% H_2_O_2_ (v/v) for 20 min. The sections were placed in 0.01 M citrate buffer and heated in a microwave oven at 95 °C for 20 min after washed with PBS. Brain sections were stained with hematoxylin and eosin (H&E), followed by dehydration, hyalinization, fixation, and observed under a high-magnification optical microscope (Olympus, Japan). Neuronal damage was evaluated on a scale of 0 = normal, 1 = a few (< 30%) neurons damaged, 2 = many (30 to 70%) neurons damaged, and 3 = majority of neurons (> 70%) damaged [25].

### Immunofluorescence staining

Mouse brain sections or cultured neurons were fixed in 4% paraformaldehyde and washed with PBS three times. Antigen retrieval was performed with citrate buffer (pH 7.0). The samples were then permeabilized with 0.1% Triton X-100 for 5 min on ice, blocked in PBS containing 10% normal goat serum at room temperature for one hour, and washed three times with PBS. Brain sections and cultured neurons were incubated with primary antibodies overnight at 4 °C. After washing with PBS, the samples were incubated with secondary antibodies in blocking buffer for 1 h. Subsequently, the cellular nucleus was stained with DAPI (1:1000, Abcam) for 7 min at room temperature. Samples were washed with wash buffer three times, and images were acquired using a confocal microscope (Olympus, Japan).

The primary antibodies used for immunostaining included NeuN (rabbit, 1:300; Abcam), MAP2 (rabbit, 1:500, Abcam), Tau46 (mouse, 1:200, Millipore), Aβ (rabbit, 1:300, Abcam), PSD95 (1:500, Abcam), and synaptophysin (1:500, Abcam). The samples were then incubated with 488/564/647 goat anti-rabbit/mouse secondary antibodies (1:250; Invitrogen).

### Golgi staining

Golgi staining was performed using the FD Rapid Golgi Stain system (FD Neuro Technologies) according to the manufacturer’s instructions. Briefly, freshly dissected brains were immersed in the impregnation solution and stored at room temperature in the dark for two weeks. Brains were then transferred to solution C and stored at 4 °C for 48 h in the dark. Brains were coronally sectioned at a thickness of 200 μm with a freezing microtome (Thermo Scientific, USA). Brain sections were stained with solution D and E. Golgi-stained neurons and dendritic segments in the cortex and hippocampal regions were observed using an Olympus confocal microscope. Dendritic branching and spine density were detected using NIH Image J software.

### Neuron OGD model and functional analysis

#### Isolation of primary neurons

Primary neurons were cultured from C57BL/6 mouse embryos (aged 5–8 days). Briefly, brain tissues were dissected from mouse pups, and the meninges were removed from the brain tissue and dissociated by enzymatic digestion. Isolated primary neurons were plated onto poly-D-lysine-coated dishes and cultured in neurobasal medium supplemented with 2% B27 (Gibco), 0.5 mM of glutamine, 10% fetal bovine serum (Gibco), and 1% penicillin/streptomycin (Sigma) and maintained in a 5% CO_2_ incubator at 37 °C. The media was changed every three days.

### OGD

The neuron oxygen–glucose deprivation (OGD) model was established according to a previously publication with minor modifications [26]. Briefly, the cultured primary neurons were washed and incubated with glucose-free DMEM (Gibco, USA) and incubated in an anaerobic chamber (Thermo Fisher Scientific, USA) containing 1% O_2_, 5% CO_2_, and 94% N_2_ for 2 h. After incubation, the culture medium was replaced with normal medium in a normoxic condition. During reoxygenation time, neurons were co-cultured with various MSC EX (EX^Con^, EX^miR-132-3p^) or culture medium (vehicle). To explore the underlying mechanism, neurons were pretreated with a Ras inhibitor (NSC 23766, 100 mM; Sellectkchem) for 2 h.

### Neuron functional analysis

Neuron viability was measured using the CCK-8 assay kit according to the manufacturer’s instructions. Neuronal apoptosis was analyzed using the Annexin V-PE/7-AAD apoptosis detection kit (BD Bioscience, USA) based on the manufacturer’s instructions followed by flow cytometric analysis. Intracellular ROS production by neurons was measured by dihydroethidium (DHE, Beyotime, China) staining and visualized under a confocal microscope (Olympus, Japan).

### Target prediction for miR-132-3p

Bioinformatics prediction of target genes and miR-132-3p binding sites was performed using different databases: TargetScan (http://www.targetscan.org/vert_72/), miRbase (https://www.mirbase.org/) and miRanda (http://www.microrna.org/). An overlapping target molecule for miR-132-3p from these databases was RASA1, which is a validated target of miR-132-3p [27], and was considered for further experimental analysis.

### Western blot analyses

The neurons were homogenized in lysis buffer including proteinase and phosphate kinase inhibitors (Sigma, USA). Proteins (30 μg) were mixed with loading buffer and electrophoresed through SDS-PAGE gels and transferred to polyvinylidene difluoride membranes (PVDF, Millipore Corporation, USA). After blocking non-specific antigens in 5% non-fat milk, the membranes were incubated with primary antibodies against RASA1 (1:1000, CST, USA), Ras (1:1000, Abcam, USA), Akt and phospho-Akt (1:500, CST), GSK-3β and phospho-GSK-3β^ser9^ (1:1000, Abcam), p-Tau (1:1000, Abcam), and CD63 (1:400, Abcam). The bands were visualized using an ECL kit (Amersham, Sweden).

### Statistical analysis

All data are expressed as mean ± SEM. Multiple comparisons were analyzed by one-way ANOVA or Student’s *t* test. GraphPad Prism 7 software was used for analyzing the data. For all measurements, a *p* < 0.05 was considered statistic significant.

## Results

### Infusion of EX^miR-132-3p^ can restore cortical and hippocampal MSC EX and miR-132-3p in VD mice

**Fig. 1:**
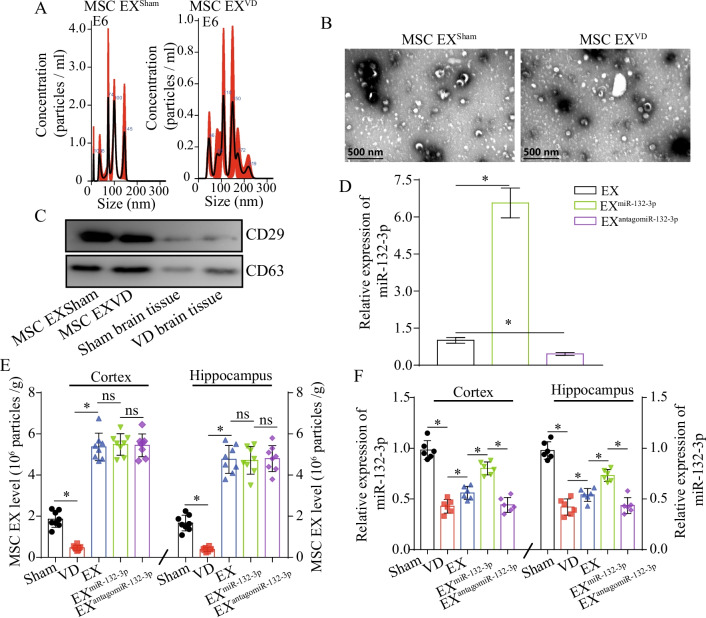
EX^miR-132-3p^ infusion restores cortical and hippocampal MSC EX and miR-132-3p level in VD mice. **A**–**C** MSC EX from sham and VD mouse brain tissue was detected by NTA, TEM, and western blotting. **D** The effects of EX^Con^/EX^miR-132-3p^/EX^antagomiR-132-3p^ infusion on cortical and hippocampal MSC EX level in Sham and VD mice. **E** The effects of EX^Con^/EX^miR-132-3p^/EX^antagomiR-132-3p^ infusion on cortical and hippocampal miR-132-3p level in Sham and VD mice. Data represent the mean ± SEM, *n* = 8 mice per group. *p < 0.05

NTA and TEM analyses showed that the MSC EX^Sham^ and MSC EX^VD^ were 100 ± 50 nm in size (Fig. 1A, B). By using western blotting assay, we verified that both of the MSC EX^Sham^ and MSC EX^VD^ were positive for CD63 and CD29 (Fig. 1C). These data indicate MSC EX was successfully isolated from mouse brain. Twenty-one days following VD surgery, cortical and hippocampal MSC EX levels were detected by combining microbeads with NTA. The level of the MSC EX was decreased by 78% in the cortex and 85% in the hippocampus of VD mice at 21 days following VD surgery compared with that of the sham group (vs. sham; *p* < 0.05; Fig. 1E). qRT-PCR analysis revealed that the level of miR-132-3p was decreased 72.9% in the cortex and 75.6% in the hippocampus at 21 days after VD surgery, compared with that of the sham group (vs. sham group; *p* < 0.05; Fig. 1F). These data suggest that the reduction of miR-132-3p and MSC EX in the cortex and hippocampus was highly correlated with VD injury.

We did experiment to evaluate the miR-132-3p level in EX after the transfection of lentivirus carrying the murine miR-132-3p or murine antago miR-132-3p. We found that miR-132-3p was significantly increased in EX^miR-132-3p^ compared to EX^Con^ (*p* < 0.05; Fig. 1D), while significantly decreased in EX^antagomiR-132-3p^ (*p* < 0.05; Fig. 1D). At day 21 after EX^Con^/EX^miR-132-3p^/EX^antagomiR-132-3p^ infusion, we observed that EXs, EX^miR-132-3p^, and EXs^antagomiR-132-3p^ significantly increased MSC EX levels in the cortex and hippocampus of VD mice (vs. Sham, *p* < 0.05; Fig. 1F). Meanwhile, we also observed that there were no significant differences between the three treatments (EX^Con^ vs. EX^miR-132-3p^ or EX^miR-132-3p^ vs. EX^antagomiR-132-3p^, *p* > 0.05; Fig. 1F). Using qRT-PCR analysis, we found that both EX and EX^miR-132-3p^ increased miR-132-3p levels in the cortex and hippocampus of VD mice (vs. PBS, *p* < 0.05; Fig. 1F) and EX^miR-132-3p^ displayed better efficacy at increasing miR-132-3p levels compared with EX-treatment (vs. EX, *p* < 0.05; Fig. 1F) and EX^antagomiR-132-3p^ decreased miR-132-3p levels compared with EX^miR-132-3p^ (vs. EX^miR-132-3p^, *p* < 0.05; Fig. 1F). These results suggest that EX^Con^/EX^miR-132-3p^/EX^antagomiR-132-3p^ infusion can restore attenuated MSC EX levels in the cortex and hippocampus of VD mice, and EX^miR-132-3p^ can increase cortical and hippocampal miR-132-3p via transferring their contained miR-132-3p.

### MiR-132-3p mediates EX-induced restoration of cognitive and synaptic functions in VD mice

**Fig. 2:**
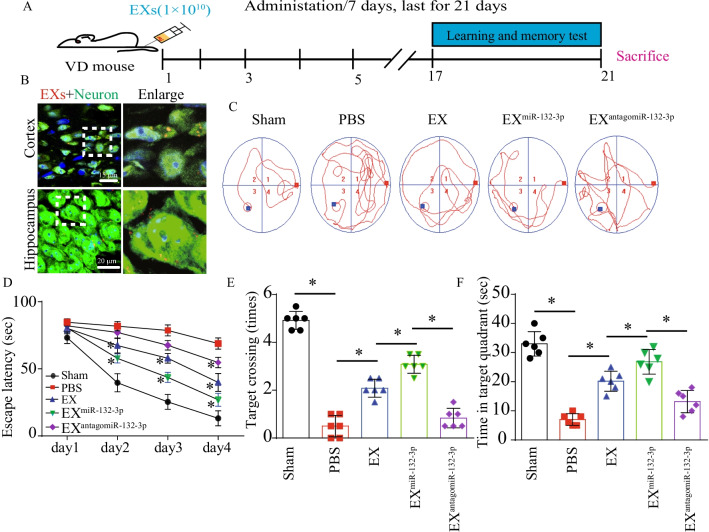
EX^miR-132-3p^ infusion rescues cognitive impairment and synaptic deficits in VD mice. **A** Workflow of MSC EX injection/rescue experiments. **B** Images showing that injected EX (PKH 26, red) merged into cortical and hippocampal neurons (NeuN, green). **C**–**F** The effects of EX injection on cognitive impairment in VD mice in MWM tests. Data represent the mean ± SEM. Three independent experiments were performed (*n* = 3 mice per group). *p < 0.05

**Fig. 3:**
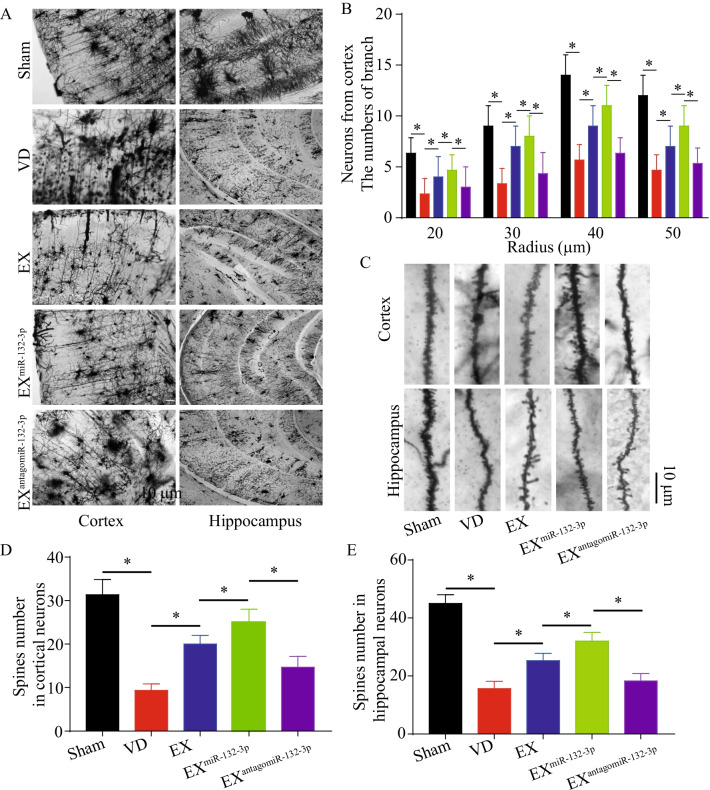
EX^miR-132-3p^ infusion rescues synaptic deficits in VD mice. **A** Representative Golgi staining from cortex and hippocampus regions are shown. Scale bar, 10 μm. **B** The effects of MSC EX injection on cortical dendritic arborization in Sham and VD mice. **C**–**E** Representative images and summary data of dendritic spines from cortical and hippocampal neurons from VD mice after treated with EX^Con^/EX^miR-132-3p^/EX^antagomiR-132-3p^. Data represent the mean ± SEM. *n* = 20 neurons from 3 mice per group. Three independent experiments were performed. *p < 0.05

As shown in Fig. 2B, we confirmed the fluorescent of PKH26-labeled MSC EX was observed in cortical and hippocampal neurons in VD mice, suggesting that the injected MSC EX merged into the cortical and hippocampal neurons. To test whether MSC EX and their carried miR-132-3p could rescue synaptic deficits, learning, and memory impairment in VD mice, EX^Con^/EX^miR-132-3p^/EX^antagomiR-132-3p^ were injected into VD mice intravenously every 7 days for 21 days. We observed that both EX and EX^miR-132-3p^ rescued escape latency deficits during training and improved time spent in the target quadrant and numbers to cross the platform in MWM tests (vs. PBS, *p* < 0.05; Fig. 2C–F), with a better effect observed with EX^miR-132-3p^ (vs. EX^Con^, *p* < 0.05; Fig. 2C–F), whereas EX^antagomiR-132-3p^ decreased cognitive function restoration compared with EX^miR-132-3p^-treated group (vs. EX^miR-132-3p^, *p* < 0.05; Fig. 2C–F). In addition, synaptic spines and dendritic complexity compromised in cortex and hippocampus of VD mice were also restored by EX^Con^ infusion (vs. PBS, *p* < 0.05; Fig. 3A–E). Similarly, infusion of EX^miR-132-3p^ significantly induced an increase of synaptic spines and dendritic complexity compared with EX-treated group (vs. EX^Con^, *p* < 0.05; Fig. 3A–E) and EX^antagomiR-132-3p^ decreased cortical and hippocampal synaptic spines and dendritic complexity compared with EX^miR-132-3p^ (vs. EX^miR-132-3p^, *p* < 0.05; Fig. 3A–E). These data indicated that miR-132-3p enrichment enhances the effects of EX on restoring cognitive and synaptic functions in VD mice.

### MiR-132-3p mediates MSC EX-induced reduction of cortical and hippocampal neuronal damage, Aβ production, and Tau hyperphosphorylation in VD mice

**Fig. 4:**
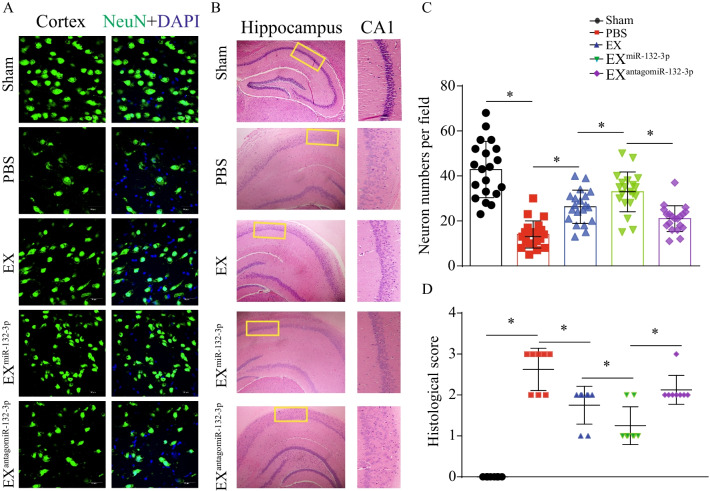
EX^miR-132-3p^ infusion reduces cortical neuron loss and abnormal architecture in hippocampal CA1 region in VD mice, injected with various MSC EX. **A**, **C** The cortical neuron number in Sham and VD mice was detected by immunofluorescence staining (NeuN, green; DAPI, blue). **B**, **D** Representative images and summary data showing the hippocampal neuron number and architecture in VD mice by immunochemistry. Data represent the mean ± SEM. *n* = 20 fields from 3 mice per group. Three independent experiments were performed. *p < 0.05

**Fig. 5:**
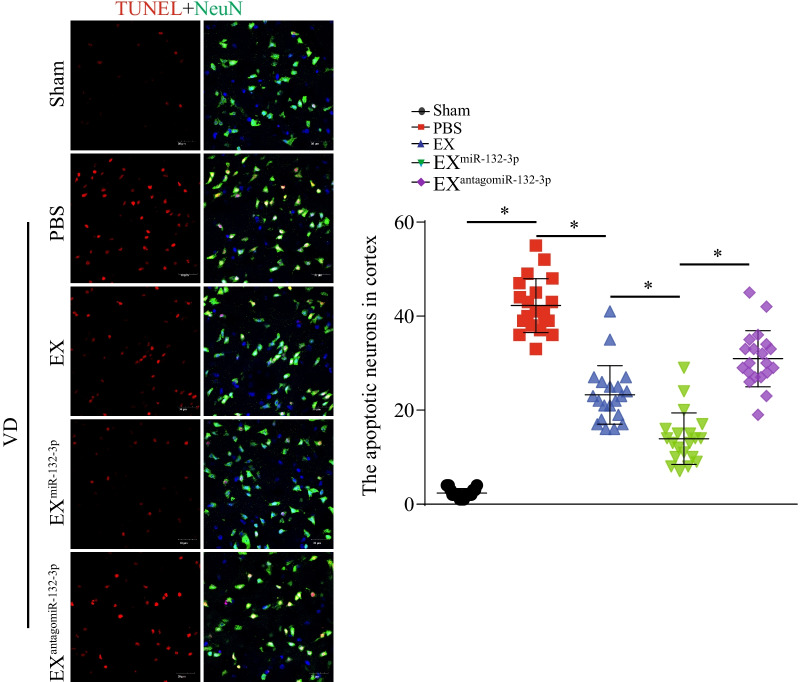
EX^miR-132-3p^ infusion reduces cortical neuron apoptosis in VD mice. The apoptotic neurons in cortex were detected by immunofluorescence staining (TUNEL, red; NeuN, green). Scale bar, 30 μm. Data represent the mean ± SEM. *n* = 20 fields from 3 mice per group. Three independent experiments were performed. *p < 0.05

To test the effects of MSC EX and their carried miR-132-3p on cortical and hippocampal neuron damage in VD mice, immunochemistry and immunofluorescence were performed in brain sections day 21 after carotid artery ligation. Compared with the sham group, VD mice showed evident neuronal cell loss and abnormal architecture in the hippocampal CA1 region, concomitant with increased neuronal cell apoptosis in the cortex (vs. sham, *p* < 0.05; Fig. 4A–D). EX^Con^ and EX^miR-132-3p^ infusion significantly reduced neuronal cell loss and abnormal architecture in the hippocampal CA1 region in VD mice (vs. PBS, *p* < 0.05; Fig. 4A–D). To our expectation, EX^miR-132-3p^ displayed better efficacy on reducing neuronal cell loss and abnormal architecture compared with EX-treated group (vs. EX^Con^, *p* < 0.05; Fig. 4A–D) and EX^antagomiR-132-3p^ showed attenuated effect on reducing cortical and hippocampal neuron damage (vs. EX^miR-132-3p^, *p* < 0.05; Fig. 4A–D). Additionally, EX^Con^ infusion significantly decreased cortical neuron apoptosis (vs. PBS, *p* < 0.05; Fig. 5). EX^miR-132-3p^ significantly decreased cortical neuron apoptosis compared with EX^Con^ (vs. EX^Con^, *p* < 0.05; Fig. 5), and EX^antagomiR-132-3p^ showed attenuated effect on decreasing cortical neuron apoptosis (vs. EX^miR-132-3p^, *p* < 0.05; Fig. 5).

**Fig. 6:**
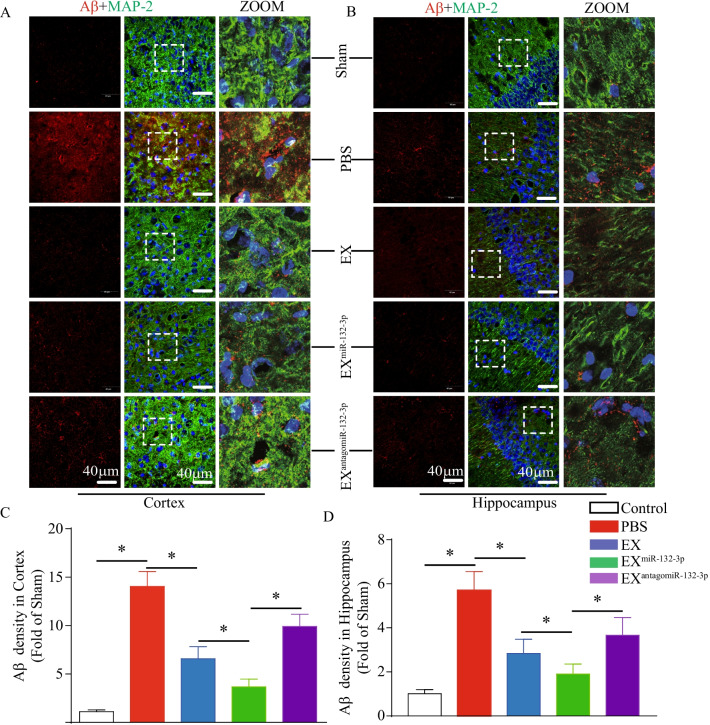
EX^miR-132-3p^ infusion reduces cortical and hippocampal Aβ production in VD mice. **A**, **C** The Aβ production in cortex was detected by immunofluorescence staining (Aβ, red; MAP-2, green). Scale bar, 40 μm. **B**, **D** The Aβ production in hippocampus was detected by immunofluorescence staining (Aβ, red; MAP-2, green). Scale bar, 40 μm. Data represent the mean ± SEM. *n* = 20 fields from 3 mice per group. Three independent experiments were performed. *p < 0.05

**Fig. 7:**
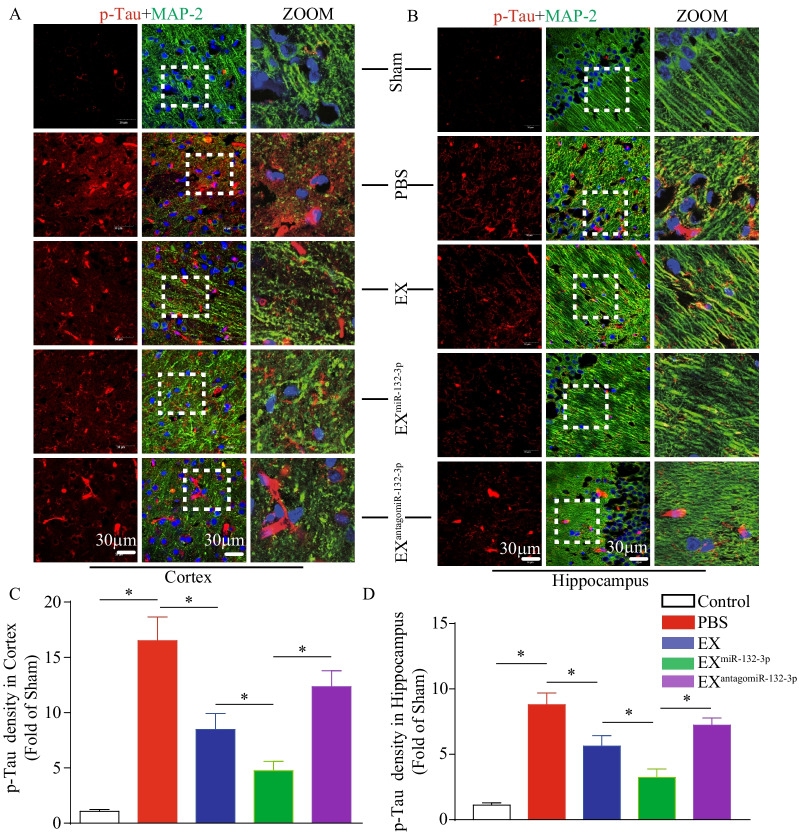
EX^miR-132-3p^ infusion reduces cortical and hippocampal Tau hyperphosphorylation in VD mice. **A**, **C** The Tau hyperphosphorylation in cortex was detected by immunofluorescence staining (p-Tau, red; MAP-2, green). Scale bar, 40 μm. **B**, **D** The Tau hyperphosphorylation in hippocampus was detected by immunofluorescence staining (p-Tau, red; MAP-2, green). Scale bar, 40 μm. Data represent the mean ± SEM. *n* = 20 fields from 3 mice per group. Three independent experiments were performed. *p < 0.05

As shown in Figs. 6 and 7, Aβ production and Tau phosphorylation in the cortex and hippocampus were increased in VD mice (vs. sham, *p* < 0.05). According to our “treatment” study, we observed that infusion of EX^Con^ and EX^miR-132-3p^ significantly decreased cortical and hippocampal Aβ production and Tau hyperphosphorylation (vs. Sham, *p* < 0.05; Figs. 6 and 7). Similarly, EX^miR-132-3p^ was more effective than EX in reducing Aβ production and Tau phosphorylation compared with EX^miR-132-3p^ and EX^antagomiR-132-3p^ showed attenuated effect on reducing Aβ production and Tau phosphorylation (vs. EX^miR-132-3p^, *p* < 0.05; Figs. 6 and 7).

Taken together, these results suggest that miR-132-3p enrichment enhances the effects of MSC EX on reducing neuronal cell damage, Aβ overproduction, and Tau hyperphosphorylation in the cortex and hippocampus of VD mice.

### MiR-132-3p mediates MSC EX-induced activation of the Ras/Akt/-GSK-3β signaling pathway in OGD-injured neurons

**Fig. 8:**
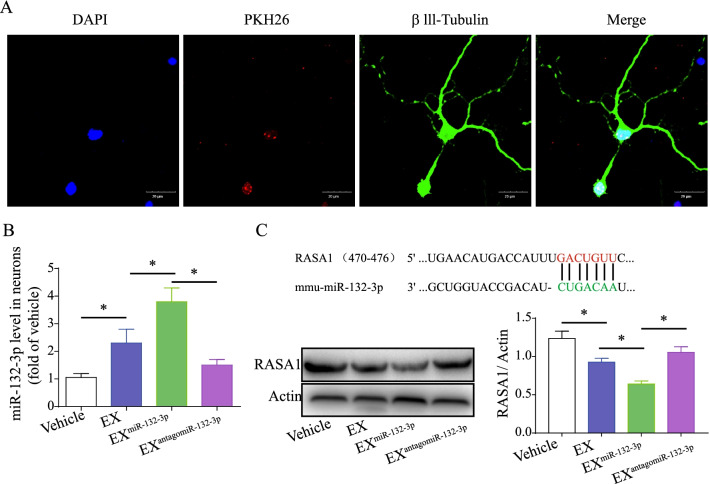
EX^miR-132-3p^ incubation increases miR-132-3p level and down-regulated the target protein RASA1 expression in OGD-treated neurons. **A** Immunofluorescence of MSC EX merged with neurons after incubation (MSC EX, red; β111-tubulin, green). Scale bar, 20 μm. **B** MiR-132-3p level in OGD-treated neurons was measured by qRT-PCR. **C** MiR-132-3p regulates the target protein RASA1 expression in OGD-treated neurons. Data represent the mean ± SEM. Three independent experiments were performed. *p < 0.05

After co-culture for 24 h, PKH26-labeled MSC EX was detected in the cytoplasm of primary neurons (Fig. 8A), suggesting that EX had merged with the neurons. Using qRT-PCR, we found that EX^Con^ and EX^miR-132-3p^ incubation significantly increased miR-132-3p expression in recipient neurons (vs. vehicle, *p* < 0.05; Fig. 8B). EX^miR-132-3p^ was more effective at increasing miR-132-3p levels compared with EX^Con^-treated group and EX^antagomiR-132-3p^ significantly decreased miR-132-3p levels in targeted neurons (vs. EX^miR-132-3p^, *p* < 0.05; Fig. 8B). These data indicate that miR-132-3p can be transferred from MSC to neurons by EX^miR-132-3p^.

**Fig. 9:**
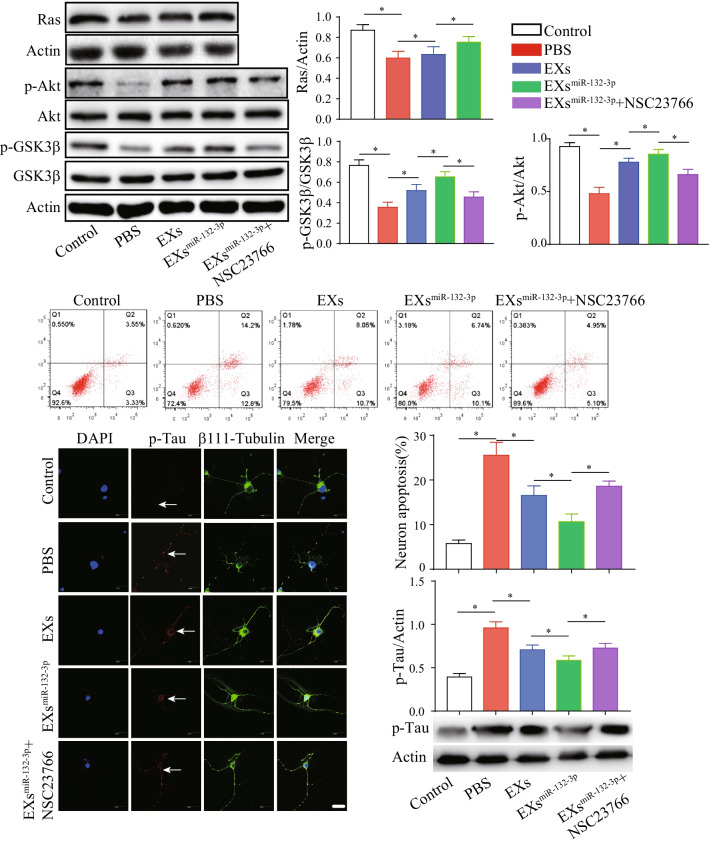
EX^miR-132-3p^ incubation decreasing neuron apoptosis and tau phosphorylation by activating Ras/Akt/GSK-3β signaling pathway in OGD-treated neurons. **A**, **B** Representative images and summary data showing the level of Ras and phosphorylation of Akt and GSK-3β in OGD-treated neurons after various MSC EX incubation. **C** The neuron apoptosis was detected by flow cytometry. **D**, **E** Representative images and summary data showing the p-Tau level in OGD-treated neurons after various MSC EX incubation (p-Tau, green; nucleus, blue). Scale bar, 40 μm. **F** The phosphorylation of Tau in OGD-treated neurons after various MSC EX incubation was measured by western blotting. Data represent the mean ± SEM. Three independent experiments were performed. *p < 0.05

Using bioinformatics analysis, we found RASA1 is a predicted target of miR-132-3p (Fig. 8C). As expected, we found that RASA1 expression in OGD-treated neurons was significantly decreased after EX^Con^ incubation (vs. vehicle, *p* < 0.05; Fig. 8C) and EX^miR-132-3p^ exhibits greater effects on decreasing RASA1 expression (vs. EX^Con^, *p* < 0.05; Fig. 8C). The downstream Ras, Akt, p-Akt, GSK-3β, and p-GSK-3β^ser9^ proteins levels were also examined by western blotting assay. We found that OGD treatment significantly decreased Ras expression and the phosphorylation of Akt and GSK-3β in neurons (vs. control, *p* < 0.05; Fig. 9A, B). EX incubation increased Ras and the phosphorylation of Akt and GSK-3β in OGD-treated neurons (vs. vehicle, *p* < 0.05; Fig. 9A, B). Again, EX^miR-132-3p^ was more effective in increasing Ras and the phosphorylation of Akt and GSK-3β compared with EX^Con^-treated group and Ras inhibitor (NSC23766) partially abolished the effects of EX^miR-132-3p^ on increasing Ras levels and phosphorylation of Akt and GSK-3β in OGD-treated neurons (vs. EX^miR-132-3p^, *p* < 0.05; Fig. 9A, B). These results suggest that miR-132-3p enrichment enhances the effects of MSC EX on activating the Ras/Akt/GSK-3β signaling pathway by targeting RASA1 in OGD-treated neurons.

### MiR-132-3p mediates EX-induced decreasing of Tau hyperphosphorylation and apoptosis in OGD-injured neurons by activating the Ras/Akt/-GSK-3β signaling pathway

As the role of EXs is highly correlated with the status of the source cells, we carried out experiment to analyze the effects of miR-132-3p in MSC proliferation and senescence. We found miR-132-3p overexpression increased the proliferation of MSC (MSC^miR-132-3p^ vs. MSC^Con^, *p* < 0.05; Additional file 1: Fig. S1A), while knockdown miR-132-3p decreased the proliferation ability of MSC (MSC^antagomiR-132-3p^ vs. MSC^miR-132-3p^, *p* < 0.05; Additional file 1: Fig. S1A). Additionally, we observed that there was no significance in senescence between MSC^miR-132-3p^ and MSC^antagomiR-132-3p^ (MSC^miR-132-3p^ vs. MSC^antagomiR-132-3p^, *p* > 0.05; Additional file 1: Fig. S1B). Our results demonstrated that miR-132-3p could improve MSC activation, suggesting a beneficial role of miR-132-3p in MSC EX function. As shown in Fig. 9C–F, we observed that OGD-treated neurons exhibited a marked increase in Tau phosphorylation, and apoptosis (vs. sham, *p* < 0.05), and EX^Con^ incubation decreased Tau phosphorylation and apoptosis in OGD-treated neurons (vs. vehicle, *p* < 0.05; Fig. 9C–F). Similarly, EX^miR-132-3p^ exhibited a greater beneficial effect compared with EX^Con^-treated group and NSC23766 partially abolished the effects of EX^miR-132-3p^ on decreasing Tau phosphorylation and apoptosis in OGD-treated neurons (vs. EX^miR-132-3p^, *p* < 0.05; Fig. 9C–F). These results indicate that miR-132-3p enrichment promotes the effects of MSC EX on reducing Tau hyperphosphorylation and apoptosis in OGD-treated neurons by activating the Ras/Akt/-GSK-3β signaling pathway.

### MiR-132-3p mediates EX-induced increasing of neurite outgrowth and synaptic density in OGD-injured neurons by activating the Ras/Akt/-GSK-3β signaling pathway

**Fig. 10:**
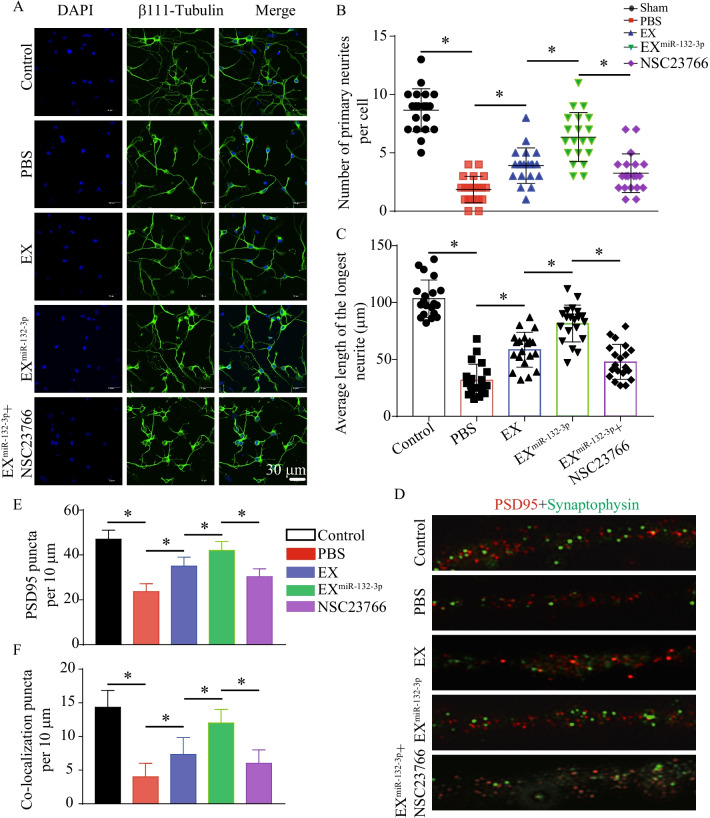
EX^miR-132-3p^ incubation restores neurite outgrowth and synaptic density in OGD-treated neurons. **A**–**C** Representative images and summary data showing neurite outgrowth in OGD-treated neurons after various MSC EX incubation (β111-tubulin, green; nucleus, blue). Scale bar, 30 μm. **D**–**F** Representative images and summary data showing synaptic density in OGD-treated neurons after various MSC EX incubation (PSD95, red; synaptophysin, green). Data represent the mean ± SEM. Three independent experiments were performed. *p < 0.05

To investigate the effect of MSC EX and their carried miR-132-3p on neurite outgrowth in OGD-treated neurons, EX^Con^/EX^miR-132-3p^ were co-cultured with OGD-injured neurons for 24 h. The neurite branch number and total neurite length were compromised in OGD-treated neurons, but were ameliorated by EX^Con^ incubation (vs. vehicle, *p* < 0.05; Fig. 10A–C). Again, EX^miR-132-3p^ was more effective than EX^Con^ in improving neurite branch number and total neurite length in OGD-injured neurons (vs. EX^Con^, *p* < 0.05; Fig. 10A–C). Pretreatment with NSC23766 partially abolished the effects of the EX^miR-132-3p^ (vs. EX^miR-132-3p^, *p* < 0.05; Fig. 10A–C).

To determine whether MSC EX and their carried miR-132-3p could promote synaptic density in OGD-injured neurons, we analyzed the expression of synaptophysin (synapsin 1) and postsynaptic density protein (PSD95) by immunofluorescence staining. We observed that EX incubation significantly increased synapsin 1 and PSD95 expression, which were compromised in OGD-treated neurons (vs. Sham, *p* < 0.05; Fig. 10D–F), and EX^miR-132-3p^ displayed better efficacy. Furthermore, pretreatment with NSC23766 partially abolished the effects of the EX^miR-132-3p^ on increasing synapsin 1 and PSD95 expression in OGD-treated neurons (vs. EX^miR-132-3p^, *p* < 0.05; Fig. 10D–F).

Taken together, these results indicate that miR-132-3p enrichment enhances the effects of MSC EX on improving neurite outgrowth and synaptic density in OGD-injured neurons.

## Discussion

This study demonstrates that infusion of miR-132-3p-enriched MSC EX improves synaptic and cognitive function in VD mice by increasing miR-132-3p and MSC EX levels, but decreasing Aβ aggregation, Tau hyperphosphorylation, and neuron damage in the cortex and hippocampus. Furthermore, in vitro mechanistic studies have found miR-132-3p priming enhanced the effects of MSC EX on protecting cells from OGD-induced oxidative stress, apoptosis, and synaptic function disruption by targeting RASA1 and activating the downstream Ras/Akt/GSK-3β signaling pathway in primary cultured neurons.

Ischemia-induced neuron damage as well as dendritic and synaptic abnormalities can lead to neuronal dysfunction, which is the primary cause of cognitive impairment in VD [1]. MSCs are known as “sentinel and safe-guards of injury” and promote neuroprotection in various neurological diseases, including AD, ischemic stroke (IS), and vascular cognitive impairment (VCI) [28]. It was found that the neuroprotective effects of MSC depended primarily on exosome-mediated paracrine rather than cell replacement [29]. In this study, we firstly observed that MSC EX levels were significantly decreased in the brain tissue of VD mice, concomitant with cognitive impairment. Additionally, our data showed an impaired endogenous protective effect of MSC EX during VD injury and along with other groups, indicating that systemic administration of MSC EX could improve neuronal function in an IS animal model, which is consistent with the findings of other studies [13, 19]. In the AD mice model, infusion of MSC EX ameliorates learning and memory deficits by decreasing Aβ accumulation and restoring synaptic function [11]. However, to our knowledge, the therapeutic effects of MSC EX in VD are still unknown. Therefore, MSC EX in the present study was systemically administrated to VD mice via the tail vein, which entered the brain and restored MSC EX levels in the hippocampus and cortex to explore the specific mechanism of MSC EX in VD mice. Interestingly, MSC EX infusion improved learning and memory in the VD mice, which indicate a therapeutic effect of MSC EX in VD. Since the regulation of neuronal density, spine formation, and synaptic plasticity are critical in maintaining normal cognitive function [30, 31], we here evaluated the effect of MSC EX infusion on neuronal cell density and architecture, spine formation, and synaptic plasticity in the hippocampus and cortex by using H&E staining and immunofluorescence. As expected, MSC EX infusion rescued VD-induced neuronal cell loss and abnormal architecture [22], spine formation, and synaptic plasticity impairment [32]. Our findings provide new evidence regarding the therapeutic efficacy of MSC EX in cognitive decline [11]. However, we do not exclude the possibility that the miR-132-3p-enriched MSC EX treatment also merged into cerebral endothelial cells, astrocytes, and microglia in cortex and hippocampus, which also may contribute to protect neuron dysfunction and cognitive impairment from VD.

EX can regulate target cell function by delivering their cargos (miRs, proteins, and lipids) obtained from source cells and miRs are important functional cargos [33]. MiR-132 is one of the most abundant miRs in the brain, where it regulates neuronal function and promotes synaptic plasticity [34]. In addition, a decrease in miR-132 expression was found in postmortem AD [35] and was associated with impaired cognitive function [36]. In the present study, we found that the level of miR-132-3p is obviously decreased in the hippocampus and cortex of VD mice, suggesting that miR-132-3p plays a role in VD; however, little is known about the protective effects of MSC EX enriched with miR-132-3p. Based on this evidence, we transfected MSC with miR-132-3p mimics to generate miR-132-3p-enriched MSC EX, and determined whether EX^miR-132-3p^ infusion could enhance the therapeutic effects of MSC EX on VD. EX^miR-132-3p^ infusion significantly increased the level of miR-132-3p in the hippocampus and cortex of VD mice, while EX^antagomiR-132-3p^ decreased cortical and hippocampal miR-132-3p expression compared with EX^miR-132-3p^-treatment, suggesting the effective transfer of miR-132-3p to the brain by EX^miR-132-3p^. Following intravenous administration, PKH26-labeled MSC EX crosses the blood–brain barrier and merges into neurons in the hippocampus and cortex of VD mice. We and others have shown that the systemic administrated MSC EX enriched with neurovascular protective signaling molecules, such as miRs, can be incorporated into neurovascular cells to modify their functions in the brain [6, 19]. Therefore, we further investigated the protective role of MSC EX and their carried miR-132-3p in neuronal dysfunction and cognitive impairment during VD. Our data revealed that the neuronal density, spine formation, and synaptic plasticity in the cortex and hippocampus of VD mice were further increased by the miR-132-3p-enriched MSC EX treatment. This is consistent with a previous study, which indicated miR-132 could reduce neuron death and promote neurite elongation and branching [34]. Taken together, miR-132-3p promotes the therapeutic effects of MSC EX on neuron impairment of VD. Beside exosomes, there is evidence demonstrated that MSC secretome exerted an important role in regulating target cell functions, such as senescence [37], proliferation [20], and migration [38]. Recent studies also confirmed the protective effects of MSC secretome in neural function [39, 40]. In this study, we showed that miR-132-3p could improve the effects of MSC EX in protecting neuron function. Thus, we suppose that miR-132-3p may do beneficial effects in MSC secretome in protecting neuron damage from vascular dementia. Further studies are needed to clarify the effects of MSC secretome in neuron function and vascular dementia.

We further investigated the potential mechanisms involved in the therapeutic effects of MSC EX and their miR-132-3p cargo in VD mice. The increased levels of Aβ aggregation and Tau hyperphosphorylation in brain tissue play an important role in inducing neuron damage, synaptic dysfunction, and cognitive impairment in various neurological disorders [14, 34]. Evidence shows that MSC EX may decrease both secreted and intracellular Aβ levels in N2a cells [41], and restore the Aβ-induced synaptic plasticity dysfunction in the hippocampus of AD mice [10]. In the present study, we demonstrated that MSC EX infusion significantly decreased Aβ levels and accumulation in the cortex and hippocampus of VD mice. Our findings are consistent with a previous report, demonstrating that MSC EX protects neurons from synapse damage by promoting Aβ clearance [42]. Moreover, we observed that MSC EX infusion reduced Tau hyperphosphorylation in the cortex and hippocampus of VD mice, which furtherly proved the role of MSC in reducing Tau hyperphosphorylation [43]. Recently, MiR-132-3p was found to play an important role in regulating Aβ production and Tau hyperphosphorylation. Of note, the level of Tau phosphorylation and aggregation was markedly increased in miR-132-deficient mice [14]. In the AD mouse model, deletion of miR-132 also promoted Aβ production and plaque accumulation [44]. Likewise, we demonstrated in this study that EX^miR-132-3p^ infusion was more effective at decreasing Aβ production, aggregation, and Tau hyperphosphorylation in the cortex and hippocampus in VD mice, suggesting that MSC EX and their miR-132-3p cargo exert therapeutic effects on VD by reducing Aβ production and Tau hyperphosphorylation.

GSK-3β is one of the most important protein kinases implicated in the regulation of Aβ production and Tau phosphorylation, and related to neuronal dysfunction and cognitive impairment [45]. Activation of Akt increases phosphorylation of GSK-3β at serine 9 (GSK-3β^ser9^) and then inhibits the activation of GSK-3β [46], which can enhance synaptic plasticity and cognitive function [45]. Conversely, reducing Akt phosphorylation was associated with GSK-3β activation and ultimately resulted in synaptic plasticity and memory impairment [47]. Thus, Akt/GSK-3β signaling plays a central role in regulating neuronal function and synaptic plasticity. Among the many direct target proteins involved in signaling downstream of miR-132-3p, RASA1 mediates the activity of miR-132 in neuronal function and synaptic plasticity [48]. Here, we demonstrated that miR-132-3p enhanced the effects of MSC EX in down-regulating RASA1 and up-regulating Ras, and subsequently increasing the phosphorylation of Akt and GSK-3β^ser9^, which thereby decreased Tau hyperphosphorylation in OGD-treated neurons. Using a Ras inhibitor, we further demonstrated that MSC EX with miR-132-3p enrichment increases neuron viability and neurite outgrowth, while decreasing neuronal apoptosis in OGD-treated neurons by activating the Ras/Akt/GSK-3β signaling pathway. This is consistent with our in vivo data showing decreased neuronal cell loss and synaptic dysfunction in the cortex and hippocampus of VD mice treated with EX^miR-132-3p^. Moreover, the synaptic proteins, including synapsin1 and PSD95, are critical in maintaining normal synaptic function [11]. MSC EX with miR-132-3p enrichment markedly increased synapsin 1 and PSD95 expression by activating the Ras/Akt/GSK-3β signaling pathway in OGD-treated neurons. However, since the potential targets of the miR-132-3p are vast and miR-132-3p-mediated signaling pathways are complex under VD condition, our results on the mechanism of miR-132-3p through RASA1/Ras/Akt/GSK-3β signaling pathway did not to exclude the possibility of its other target of miR-132-3p, such as heterogeneous nuclear ribonucleoprotein U (HNRNPU) [49], histone deacetylase 3 (HDAC3) [50], and mitogen-activated protein kinase kinase kinase 3 (MEKK3) [51], which may also participate in the enhancement of neuroprotective effect and synaptic plasticity of miR-132-3p-enriched MSC EX. To further confirm that the miR-132-3p-enriched MSC EX promotes neuron protection and synaptic remodeling through the Ras/Akt/GSK-3β signaling pathway, experiments inviting specific overexpressing RASA1 as well as specific knockdown of Ras, and phosphorylation of Akt and GSK-3β in the neurons is needed to be performed in vivo or in vitro.

## Conclusions

In summary, our findings suggest that MSC EX represents a promising therapeutic strategy for VD by ameliorating neuron damage, synaptic dysfunction, and cognitive impairment. Furthermore, they prevent Aβ production, aggregation, and Tau hyperphosphorylation, whereas miR-132-3p enrichment enhances these beneficial effects. Our data further demonstrate that miR-132-3p increases the protective effects of MSC EX on OGD-induced neuron apoptosis, synaptic disruption, and Tau hyperphosphorylation through activation of the Ras/Akt/GSK-3β signaling pathway.

## Supplementary Information

**Additional file 1. Fig. S1**: The effect of miR-132-3p in MSC (passage 2) proliferation and senescence. Cell proliferation of MSC was determined by Cell Counting Kit 8 (CCK-8) assay. The graph shows data coming from MSC^Con^, MSC^miR-132-3p^, and MSC^antagomiR-132-3p^ groups (A). Representative images and summary data showing the senescence of MSC in MSC^Con^, MSC^miR-132-3p^, and MSC^antagomiR-132-3p^ groups. *p＜0.05. (PDF 2433 kb)

## Data Availability

All data generated or analyzed during this study are included in this published article.

## Abbreviations

VD: Vascular dementia
MiR-132-3p: MicroRNA-132-3p
MSC: Mesenchymal stromal cells
EXs: Exosomes
OGD: Oxygen–glucose deprivation
Rasa 1: RAS p21 protein activator 1
p-Akt: Phosphorylation-Akt
PBS: Phosphate-buffered saline
CNS: Central nervous system
NTA: Nanoparticle tract analysis
MWM: Morris water maze
qRT-PCR: Quantitative real-time PCR
NTA: Nanoparticle tract analysis
H&E: Hematoxylin and eosin
DHE: Dihydroethidium
PVDF: Polyvinylidene difluoride membranes
PSD 95: Postsynaptic density protein 95
