# Supplementary figures and images for: Exosomal miR-132-3p from mesenchymal stromal cells improves synaptic dysfunction and cognitive decline in vascular dementia
Source: Stem Cell Res Ther. 2022 Jul 15;13:315. doi: 10.1186/s13287-022-02995-w (PMC9284820; doi:10.1186/s13287-022-02995-w)

A

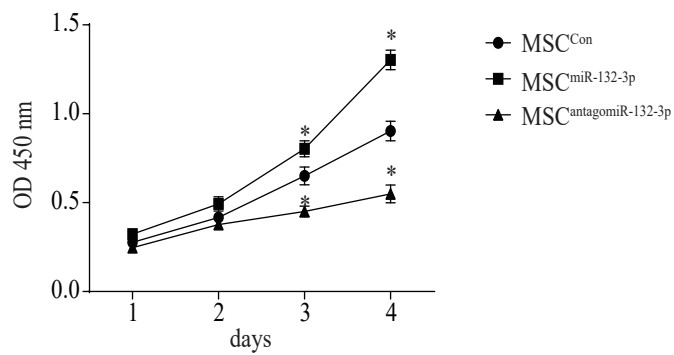

B

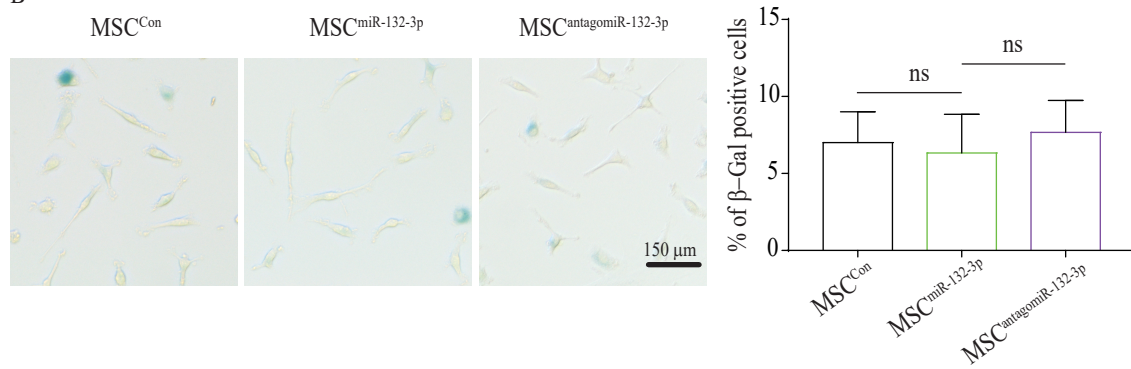

Supplement: Supplementary file 1 — Additional file 1. Fig. S1: The effect of miR-132-3p in MSC (passage 2) proliferation and senescence. Cell proliferation of MSC was determined by Cell Counting Kit 8 (CCK-8) assay. The graph shows data coming from MSCCon, MSCmiR-132-3p, and MSCantagomiR-132-3p groups (A). Representative images and summary data showing the senescence of MSC in MSCCon, MSCmiR-132-3p, and MSCantagomiR-132-3p groups. *p＜0.05. (PDF 2433 kb) [file 13287_2022_2995_MOESM1_ESM.pdf]
